# Supplementary material for: Multiple Analysis and Characterization of Novel and Environmentally Friendly Feather Protein-Based Wood Preservatives
Source: Polymers (Basel). 2020 Jan 19;12(1):237. doi: 10.3390/polym12010237 (PMC7023607; doi:10.3390/polym12010237)
Supplement: Supplementary file 1 [file polymers-12-00237-s001.pdf]

Article

Multiple Analysis and Characterization of Novel and Environmentally Friendly Feather Protein-Based Wood Preservatives

Yan Xia [1,2], Chengye Ma [1], Hanmin Wang [1], Shaoni Sun [1], Jialong Wen [1,*] and Runcang Sun [1,3,*]

[1] Beijing Key Laboratory of Lignocellulosic Chemistry, Beijing Forestry University, Beijing 100083, China; xiayan@swfu.edu.cn (Y.X.); chengye.ma@foxmail.com (C.M.); wanghanmin798@163.com (H.W.); sunshaoni@bjfu.edu.cn (S.S.)

[2] College of Material Science and Engineering, South-west Forestry University, Kunming 650224, China

[3] Center for Lignocellulose Science and Engineering, Dalian Polytechnic University, Dalian 116034, China

* Correspondence: wenjialong@bjfu.edu.cn (J.W.); rcsun3@bjfu.edu.cn (R.S.); Tel.: +86-10-62336903 (J.W. & R.S.); Fax: +86-10-62336903 (J.W. & R.S.)

Supplementary caption

1. Target retention

2. Measured retention

3. Weight percent gain (WPG)

Figure S1. Raman spectra of wood samples before and after decay.

Table S1. The cost comparison between feather protein-based preservatives and commercial wood preservatives (CCA and ACQ)

1. Target retention

Target retention means active ingredient absorbed in 1 m^3 of wood blocks by treating with the preservative.

2. Measured retention

On account of wood structure is porous and capillary, the target retention could not be completely achieved due to the air pressure presents in the wood cell wall. Consequently, the actual retention needs to be measured to determine the active ingredient actually absorbed in the treated wood blocks. Measured retention is calculated by the difference between the final wet weights after treatment and the dry weights before treatment. Treatability meant the actual percentage retention through the ratio of measured retention and target retention for the preservative.

Measured retention = $(m_1\text{-}m_0)\times\text{Contentration}_{Cu/B}$

3. Weight percent gain (WPG)

WPG means the weight gain rate of the treated wood blocks, which is calculated by the difference between the final dry weights before and after treatment, representing the actual ingredients of the preservative ultimately left in the wood.

$$\text{WPG} = \frac{m_2\text{-}m_0}{m_0}\times 100\%$$

In the formula, m_0 is the dry weight of wood block sample before immersed in the preservatives. m_1 is the wet weight of wood block sample after immersed in the preservatives without drying. m_2 is the dry weight of wood block sample after immersed in the preservatives through drying.

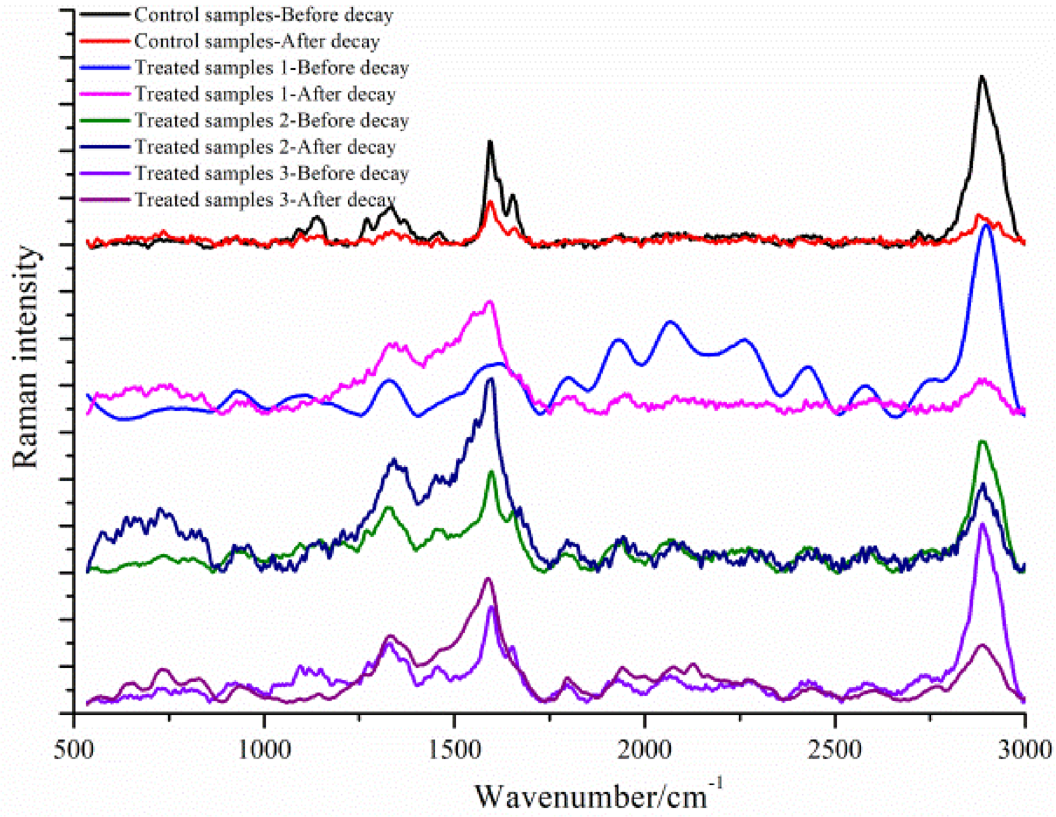
Control samples-Before decay
Control samples-After decay
Treated samples 1-Before decay
Treated samples 1-After decay
Treated samples 2-Before decay
Treated samples 2-After decay
Treated samples 3-Before decay
Treated samples 3-After decay
Raman intensity
500
1000
1500
2000
2500
3000
Wavenumber/cm-1

Fig. S1

Table S1. The cost comparison between feather protein-based preservatives and commercial wood preservatives (CCA and ACQ)

	P_1, Cu-B-Pr	P_2, Cu-B-Pr-HA	P_3, Cu-B-Pr-Go
Copper sulfate	156-180	156-180	156-180
Sodium borate	80-160	80-160	80-160
Condensed protein hydrolyzate	Free	Free	Free
Ammonium hydroxide	80-100	80-100	80-100
Nano-carrier	--	800-1200	1000-3000
Total /RMB·m^3	320-450	1250-1650	1450-3450
CCA /RMB·m^3	2000-3000	ACQ /RMB·m^3	2000-3000

The current costs of feather protein-based preservatives, CCA and ACQ are compared in the Table S1. As can be seen from Table S1, the costs of feather protein-based preservatives are obviously lower than CCA and ACQ preservatives, mainly attributed to the free feather protein, which is the industrial waste materials need to be disposed of chicken farm. In consequence from the comparison, the feather protein-based preservative is a kind of cost-competitive wood preservatives.
